# Combined Adaptive Immune Mechanisms Mediate Cardiac Injury After COVID-19 Vaccination

**DOI:** 10.1161/CIRCULATIONAHA.125.074644

**Published:** 2025-10-30

**Authors:** Silvia Fanti, Carlene Dyer, Inga Jóna Ingimarsdóttir, Daniel Harding, Guosu Wang, Antonio D’Amati, Eriomina Shahaj, Adalbjorg Ýr Sigurbergsdóttir, Helga Thórsdóttir, Oddný Brattberg Gunnarsdóttir, Stavroula Kanoni, Paul Wright, John Martin, Jamie Chorlton, Zoe Hollowood, Siggeir Fannar Brynjólfsson, Bjorn Rúnar Lúdvíksson, Egle Solito, Serena Bert, Jack M. Keane, Saidi A. Mohiddin, M. Paula Longhi, Federica M. Marelli-Berg

**Affiliations:** 1 William Harvey Research Institute, Barts and The London Faculty of Medicine and Dentistry, Queen Mary University of London, UK (S.F., C.D., D.H., G.W., E.S., S.K., E.S., S.B., J.M.K., S.A.M., M.P.L., F.M.M.-B.).; 2 Departments of Cardiology (I.J.I.), Landspitali – The National University Hospital of Iceland, Reykjavik, Iceland.; 3 Immunology (S.F.B., B.R.L.), Landspitali – The National University Hospital of Iceland, Reykjavik, Iceland.; 4 Department of Health Sciences, Faculty of Medicine, University of Iceland, Reykjavik, Iceland (I.J.I., A.Ý.S., H.T., O.B.G., S.F.B., B.R.L.).; 5 Barts Heart Centre, Barts Health NHS Trust, St Bartholomew’s Hospital, West Smithfield, London, UK (D.H., P.W., S.A.M.).; 6 Department of Translational Biomedicine and Neuroscience, University of Bari Medical School, Bari, Italy (A.D.).; 7 Anatomic Pathology Unit, Fondazione Policlinico Universitario “A. Gemelli” IRCCS, Università Cattolica S. Cuore, Roma, Italy (A.D.).; 8 The Salt Hill Charity, London, UK (J.M., J.C., Z.H.).; 9 Division of Medicine, University College London, UK (J.M.).

**Keywords:** autoimmunity, COVID-19, T-lymphocytes, vaccination

## Abstract

**BACKGROUND:**

The COVID-19 pandemic, caused by SARS-CoV-2, has led to the first approval of mRNA vaccines in humans. By producing the full-length SARS-CoV-2 Spike protein, they induce protective antiviral immunity. Acute myopericarditis (AMP) development after vaccination has repeatedly been reported; however, the pathogenesis of this complication remains elusive.

**METHODS:**

In-depth phenotyping of peripheral blood T cells was undertaken in cohorts of patients who developed AMP after mRNA vaccination, patients hospitalized for severe COVID-19, and healthy subjects with no cardiac side effects after mRNA vaccine. Validation studies were carried out using an experimental model of cardiac inflammation, in which a shared epitope elicits functional responses in patients and mice and induces AMP.

**RESULTS:**

We show that T cells from patients with AMP recognize vaccine-encoded Spike epitopes homologous to those of cardiac self-proteins. One of these epitopes, mimicking an amino acid sequence from a cardiomyocyte-expressed K^+^ channel, induced AMP in mice. When functional responses to the Kv2 were analyzed, patients with AMP after mRNA vaccination, but not patients with COVID-19, displayed an expanded pattern of cytokine production similar to that observed in AMP mice and in autoimmune myocarditis. Crucially, T-cell autoimmunity segregates to cardiotropic cMet (c-mesenchymal epithelial transition factor)–expressing T cells and is prevented by cMet inhibition, suggesting that heart homing imprinting, permitted by the unique mRNA vaccine biodistribution, is required for AMP development.

**CONCLUSIONS:**

AMP development after mRNA vaccines is mediated by distinct immune components, including molecular mimicry, T-cell receptor affinity, and, importantly, homing imprinting.

**Clinical Perspective**

**What Is New?**

- T cells from patients who developed myopericarditis after mRNA COVID vaccine recognize vaccine-encoded Spike epitopes homologous to those of cardiac self-proteins.
- One of these, mimicking a peptide sequence from a K^+^ channel expressed by cardiomyocytes, induces acute myopericarditis in susceptible mice.
- Development of T cell tropism for the heart (c-mesenchymal epithelial transition factor expression) is instrumental for disease development.

**What Are the Clinical Implications?**

- Post-mRNA vaccine myopericarditis is driven by molecular mimicry in susceptible individuals.
- mRNA vaccine biodistribution determines the occurrence of cardiac-selective autoimmunity by favoring heart homing imprinting.
- These findings provide a platform for the innovation of mRNA vaccines.

mRNA vaccines are a novel platform to effectively tackle both existing and emerging pathogens. Currently approved mRNA vaccines include only those against SARS-CoV-2 and which have shown robust immunogenicity and substantial protection against mortality and severe disease. Numerous mRNA vaccines against viral pathogens are currently being developed and trialled.^1^ Development of acute myopericarditis (AMP) has been repeatedly reported after vaccination with mRNA COVID-19 vaccines,^2–5^ the Moderna (mRNA-1273) and Pfizer/BioNTech (BNT162b2) vaccines, which encode for the full-length SARS-CoV-2 Spike protein, responsible for viral attachment and cellular infection.^6^ AMP has been reported after adenovirus vector vaccines (ChAdOx1, AstraZeneca, and Ad26.COV2.S, Janssen), also encoding the Spike protein, which have been used in European countries.^7–10^ In 2 large UK studies, an increased risk of myocarditis associated with the first dose of ChAdOx1 and BNT162b2 vaccines and the first and second doses of the mRNA-1273 vaccine and after mRNA vaccines given after a first dose of ChADOx1, over the 1- to 28-day post-vaccination period, and after previous exposure to SARS-CoV-2.^11,12^

Post-mRNA vaccine AMP is more frequent in men between 12 and 39 years of age, similar to other forms of myocarditis, with a mild clinical course and extremely rare cases of left ventricular dysfunction, heart failure, and arrhythmias.^13^ Of note, the risk of myocarditis after vaccination is lower than that of SARS-CoV-2 infection.^12^ Despite the estimated frequency at 106 cases per million vaccines, it has been suggested that this complication might be underdiagnosed, as investigations such as cardiac magnetic resonance imaging are not carried out in asymptomatic and mildly symptomatic patients.^13^

The pathogenesis of post-mRNA vaccine AMP has not been fully elucidated, but recent reports suggest cytokine-driven mechanisms in the context of an autoimmune response. Elevation of cytokines and metalloproteinases associated with an increase of circulating T cells and natural killer cells with features of cytokine-activated killers and accompanied by profibrotic macrophages has been reported.^14^ Similarly, neutralizing autoantibodies targeting the endogenous IL-1 (interleukin-1) receptor antagonist (which inhibits IL-1 signaling and inflammation), were observed in a cohort of patients with biopsy-proven post-vaccine myocarditis.^15^ The presence of autoantibodies and the typical occurrence after the second dose of vaccine indicate that “priming” of T-cell–mediated autoimmunity is a fundamental step toward disease development.^13,16^ The rare occurrence of this complication likely reflects the requirement for a concurrent immune context; this might include a genetically or hormonally driven background that predisposes to developing myocarditis after this and other triggering antigenic exposures.

Various mechanisms have been proposed by which COVID-19 mRNA vaccines induce antiheart autoimmunity.^17^ Potential leakage of mRNA vaccine into the circulation might result in vaccine-induced expression of SARS-CoV-2 Spike protein on the surface of cardiomyocytes and trigger an immunological response resulting in cardiomyocyte death.^18^ Alternatively, or in addition, molecular mimicry between the Spike protein of SARS-CoV-2 and cardiac self-antigens might trigger the activation of cross-reactive, autoreactive T cells in susceptible individuals.^19^ Upon injection into the deltoid muscle, mRNA-transfected cells produce Spike protein, which is then released in large amounts to the circulation, either as a whole protein or peptide fragments,^20^ and can therefore be delivered to tissues systemically. In addition, mRNA vaccines are endowed with intrinsic adjuvant activity,^21^ and mRNA and Spike protein have both been detected in the plasma and lymph nodes up to several weeks after vaccination,^22,23^ including in circulating exosomes.^24^ Thus, it is conceivable that, compared with traditional vaccines, which elicit adaptive immune responses in the lymph nodes draining the inoculation site, mRNA vaccines are capable of activating T cells systemically.

Despite many reports suggesting molecular mimicry as a causal link between COVID-19 vaccination and new-onset AMP, no cross-reactive T cells capable of inducing autoimmune cardiac damage have been identified to date.

In this study, we investigated recall responses to selected peptide sequences (epitopes) shared between mRNA-encoded Spike protein and cardiac antigen, which have previously been described to elicit immune activation in humans and mice, in patients with AMP after vaccine and healthy controls as well as in a cohort of patients with severe COVID-19. We show that a Spike epitope homologous to the cardiac Kv2 channel elicits strong recall T-cell activation in AMP patients and, crucially, is capable to induce the development of AMP in mice. We further define the features of this autoimmune response, which shares a unique cytokine signature and heart-homing properties with autoimmunity in nonvaccine-related acute myocarditis.

## Methods

The full experimental details are presented in the Supplemental Material.

### Study Populations

A total of 29 vaccinated patients in Iceland who had a subsequent adverse cardiac reaction, sought medical assistance at Landspitali University Hospital in Reykjavik or Akureyri Hospital in Northern Iceland or were reported to the Icelandic Medicines Agency.

After analysis of available clinical data, 11 patients fulfilled diagnostic criteria for definitive or probable myocarditis/pericarditis/myopericarditis according to Brighton Collaboration case definition criteria.^25^ Except for 1 patient, who developed this complication 2 weeks after the first dose of an mRNA vaccine, all patients in the cohort developed the complication within a week after the second and third mRNA vaccination (Table S1). Consistent with published reports, previous adenoviral vaccine was permitted in the latter 2 instances.^11,12^ The study was approved by the National Bioethics Committee in Iceland (reference VSN b2021100016/03.01). As in previous studies,^26^ cases in which pericarditis was present were only included if definitive or probable myocarditis was also present; the term AMP was therefore used to refer to the wider diagnostic category.

Details regarding patients with COVID-19 and healthy controls can be found in the Supplemental Methods.

### Animals

All animal protocols used in this study were approved by the animal use and care committee of Queen Mary University of London, following home office guidance (Scientific Procedure Act 1986) and the Guide for the Care and Use of Laboratory Animals of the National Research Council (license No. P71E91C8E to F.M.M.-B.). Experimental design and reporting were conducted following the Animal Research: Reporting of In Vivo Experiments guidelines.^27^

### Reagents

A full list of reagents is provided in Table S3.

### Statistical Analysis

In the in vivo experiments, sample size was determined by power calculations based on pilot studies. Power calculations were conducted using the following G*Power software version 3.1.9.7 setting: α=0.05, power=0.80. In the murine model, 3 healthy controls and 10 diseased animals allowed detection of a minimum effect size of 2.02 (SD units). In the human cohorts (n=8 healthy controls and n=10 patients with post-mRNA vaccination AMP [Vacc-AMP]/COVID-19), we were able to detect a minimum effect size of 1.42 (SD units).

The Kolmogorov Smirnov test was used to check for normal distribution of the mouse and human outcomes. To assess the assumption of equal variances, we performed the F test, which returned a nonsignificant result, indicating that variances were not significantly different among groups. All data are presented as median and interquartile range or median±interquartile range, as indicated in each figure and legend. The number of experiments (N) and biological replicates/animals (n) is stated in the figure legends. No samples or data points were omitted from analysis.

Unless otherwise noted, statistical comparisons were performed using paired and unpaired 2-tailed Student’s *t* test, 1-way or 2-way or 2-way mixed-effects ANOVA, followed by Dunnett test for multiple comparisons or by Tukey’s multiple comparison test, as appropriate.

Cell proliferation was assessed using the cell “proliferation modelling” module in the FlowJo flow cytometry software according to the manufacturer’s instructions. The proportion of dividing cells was determined after automatic identification of the resting, peak 0, T cells.

Statistical analysis was performed in Prism (version 8.3.0, GraphPad Software, San Diego, CA), and 2-tailed *P*<0.05 was considered statistically significant.

### Data and Material Availability

All data associated with this study are presented in the article or Supplemental Material and will be made available upon reasonable request. Deidentified patient data are listed in Tables S1 and S2.

## Results

### Intramuscular Vaccination With mRNA-1273 Induces Myopericarditis in Mice

Previous studies modelling cardiac side effects of mRNA vaccines have reported conflicting results. Li et al observed induction of multifocal myopericarditis after high-dose intravenous administrations of the Pfizer-BioNTech mRNA vaccine (BNT162b2) in female Balb/CaN mice but not after intramuscular delivery of the BNT162b2 mRNA vaccine in the hindlimbs.^28^ This was not reproduced in 12-week-old male Balb/cOlaHsd mice after intravenous administration of BNT162b2 using a 2-dose regimen with a 14-day interval between doses.^29^ Zirkenbach et al did not detect heart inflammation in A/J and C57BL/6 male or female mice after intramuscular BNT162b2 vaccination,^30^ whereas increased cardiac inflammation and cytokine production could be observed after intramuscular mRNA vaccination of C57BL/6 and Balb/C female mice only in the presence of lipopolysaccharide-induced inflammation.^31^

**Figure 1.**
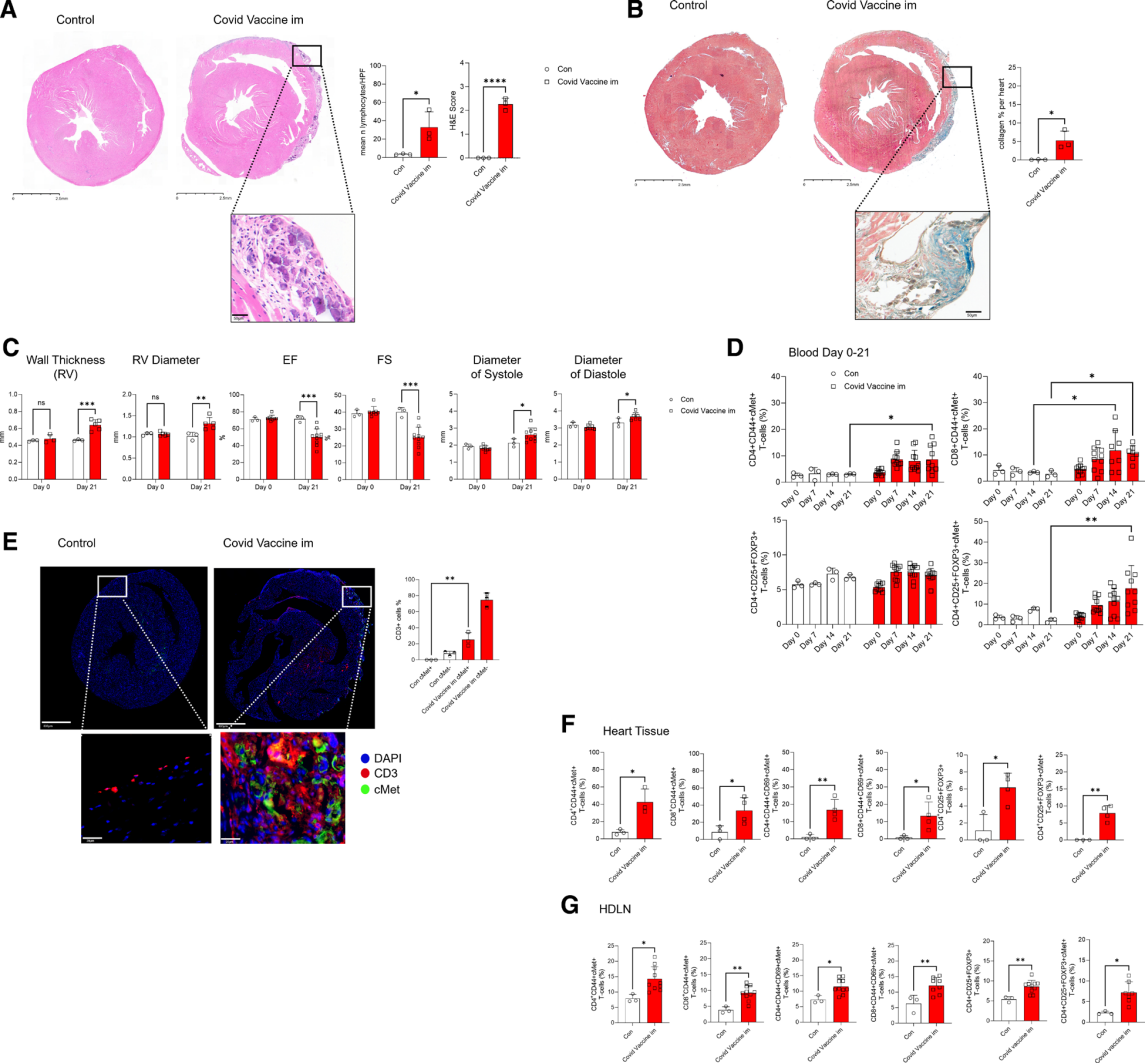
**Immunization with the mRNA-1273 vaccine induces acute myopericarditis in susceptible mice.** Five-week-old male Balb/cAnN mice received 0.25 ng/g of body weight mRNA-1273 vaccine in saline solution intramuscularly in the forelimb (COVID vaccine intramuscular) and were boosted with the same dose 14 days later. Control mice (Con) received saline solution. The development of inflammatory infiltrates and collagen deposition were assessed by hematoxylin-eosin (**A**) and Masson trichrome staining (**B**) of the heart 21 days after the first immunization. Column graphs show lymphocyte infiltration and disease scores (**A**) obtained as described in the Methods. Representative images at 10× and 40× magnification are shown on the **right** (scale bars=2.5 mm and 50 µm) Statistical analysis was performed with unpaired Student *t* test. **C**, Right ventricle (RV) wall thickness, RV diameter, left ventricle ejection fraction (EF), fractional shortening (FS), and diameter at systole and diastole were measured by echocardiography at baseline (day 0) and 21 days after the first immunization. Statistical analysis was performed with 2-way mixed-effects ANOVA followed by Tukey multiple comparison test. **D**, Tail vein blood was sampled on the same mouse at the indicated time points. The percentage of the indicated T-cell populations was determined by flow cytometry. Statistical analysis was performed with 2-way mixed-effects ANOVA followed by Tukey multiple comparison test. **E**, Immunofluorescence staining with the indicated markers was performed on heart tissue harvested on day 21. The bar graph shows the mean percentage of cMet (c-mesenchymal epithelial transition factor)^+^ and cMet^−^ T cells measured by QuPath. Statistical analysis was performed with 2-way ANOVA followed by Tukey multiple comparison test. **F** and **G**, percentage of cMet^+^ T cells measured in single-cell suspensions from whole hearts and heart-draining lymph nodes. Statistical analysis was performed with unpaired Student *t* test. All data are shown as median±interquartile range. *P<0.05, **P<0.01, ***P<0.005, ****P<0.001 (N=3, control n=3, mRNA-1273 vaccine intramuscularly n=10).

Based on these reports, we chose to use male Balb/CAnN mice, which are susceptible to myocarditis (male sex and susceptibility are also features of AMP in humans) and used intramuscular administration of the mRNA-1273 vaccine (Moderna) in the forelimb (like in humans) in a 2-dose regimen with a 14-day interval between doses. Five-week-old (youngster) Balb/cAnN male mice received 0.25 ng per gram of body weight mRNA-1273 vaccine in saline solution intramuscularly in the forelimb and were boosted with the same dose 14 days later. Control mice received an equal volume of saline solution with the same schedule. Mice were euthanized 28 days after the first vaccine inoculation. As shown in Figure 1A and 1B, immunized mice developed pathological lesions consistent with post-RNA vaccine AMP.^32^ Specifically, pericardial and subepicardial patches displayed intense and diffuse inflammatory infiltrate predominantly composed of lymphocytes and macrophages. Diseased tissue presented signs of calcification, consistent with localized intense cell death. Diffuse moderate T-cell infiltration in the deep myocardial tissue, signs of cardiomyocyte degeneration, and raised troponin levels were also observed (Figure S1A through S1D). Importantly, like in human disease,^32^ lesions were primarily localized in the right ventricle. Echocardiography also revealed functional alterations consistent with AMP, including signs of right ventricular dysfunction and consequent effects on left ventricular function (Figure 1C).^33^

We have previously described a memory T-cell subset that preferentially migrates to the heart in both mice and humans.^34^ These T cells arise upon exposure to the cytokine HGF (hepatocyte growth factor) in inflammatory conditions and upregulate the HGF receptor cMet (c-mesenchymal epithelial transition factor). Cardiotropic cMet^+^ T cells are significantly increased in the blood and the myocardium of patients with acute myocarditis as well as in mice with experimental autoimmune myocarditis.^35^ The phenotype and function of cMet^+^ T cells were distinct from those of cMet^−^ T cells, including preferential proliferation to cardiac autoantigens (cardiac myosin heavy chain [MHCα6 peptide]) and coproduction of multiple cytokines (IL-4, IL-17, and IL-22).

As shown in Figure S1E, expression of HGF was significantly increased in the lungs and heart of vaccinated animals, suggesting that the vaccine promotes systemic inflammation and production of HGF, required for cardiotropism imprinting.

We therefore monitored the proportion of circulating memory CD44^+^cMet^+^ T cells during disease onset and progression by flow cytometry (see the gating strategy in Figure S1F). Development of AMP was marked by a progressive increase of circulating CD44^+^ CD4^+^ and CD8^+^ cMet^+^ but not cMet^−^ T cells in immunized mice (Figure 1D; Figure S1G). Circulating cMet^+^ regulatory T (Treg) cells (CD4^+^ CD25^+^ FoxP3^+^) also increased, whereas total Treg cell proportions remained similar. Immunofluorescence staining of cardiac tissue from vaccinated mice showed a substantial infiltration of cMet^+^ CD3^+^ T cells in the myocardium (Figure 1E). This was confirmed when T cells infiltrating the heart were analyzed by tissue digestion and flow cytometry, which revealed increased proportions of both CD4^+^ and CD8^+^ cMet^+^ memory T cells expressing markers of recent activation (CD69 and CD25; Figure 1F). Both total and cMet-expressing Treg cells were increased in the cardiac infiltrates. Similar cMet^+^ T-cell populations were found in heart-draining lymph nodes (dLNs; Figure 1G) and spleen but not in non-dLNs (Figure S1H and S1I).

Despite limitations of the above model (dose 3 times higher than that for humans and different time lapse between dosing), the expansion of cardiotropic cMet^+^ T cells during disease development prompted us to investigate the specificity of cMet^+^ and cMet^−^ T cells in diseased animals.

To this aim, a search was conducted to identify amino acid sequence homologies between Spike and myocardium-expressed proteins. Sequences of interest were selected based on previous reports of human and murine T-cell responses to Spike-derived epitopes. Three peptides were identified and used to stimulate proliferation of CFSE (carboxyfluorescein succinimidyl ester)-labeled splenocytes from immunized and control animals. Specifically, the Spike peptide epitope P87^36–38^ is homologous to an amino acid sequence contained in Myomesin that cross-links neighboring myosin filaments and titin in the M-line of the sarcomeres.^39^ This sequence is also shared by Na^+^/Ca2^+^ exchangers (KCNC2), which play an essential role in maintaining cytosolic Ca2^+^ homeostasis.^40^ Peptide PS5^38,41^ is homologous to an amino acid sequence from the voltage-dependent K^+^ channel Kv2, which has been associated with inherited and acquired forms of long-QT syndrome.^42^ Finally, peptide P177^37,38^ is homologous to a peptide mapping to the protein Nebulette, which physically links desmin to sarcomeric actin in the heart.^43^ T cells were also challenged with the cardiac autoantigen pMHCα6 and a scrambled peptide as a control. Details of all sequences and homology with Spike are provided in the Methods section.

**Figure 2.**
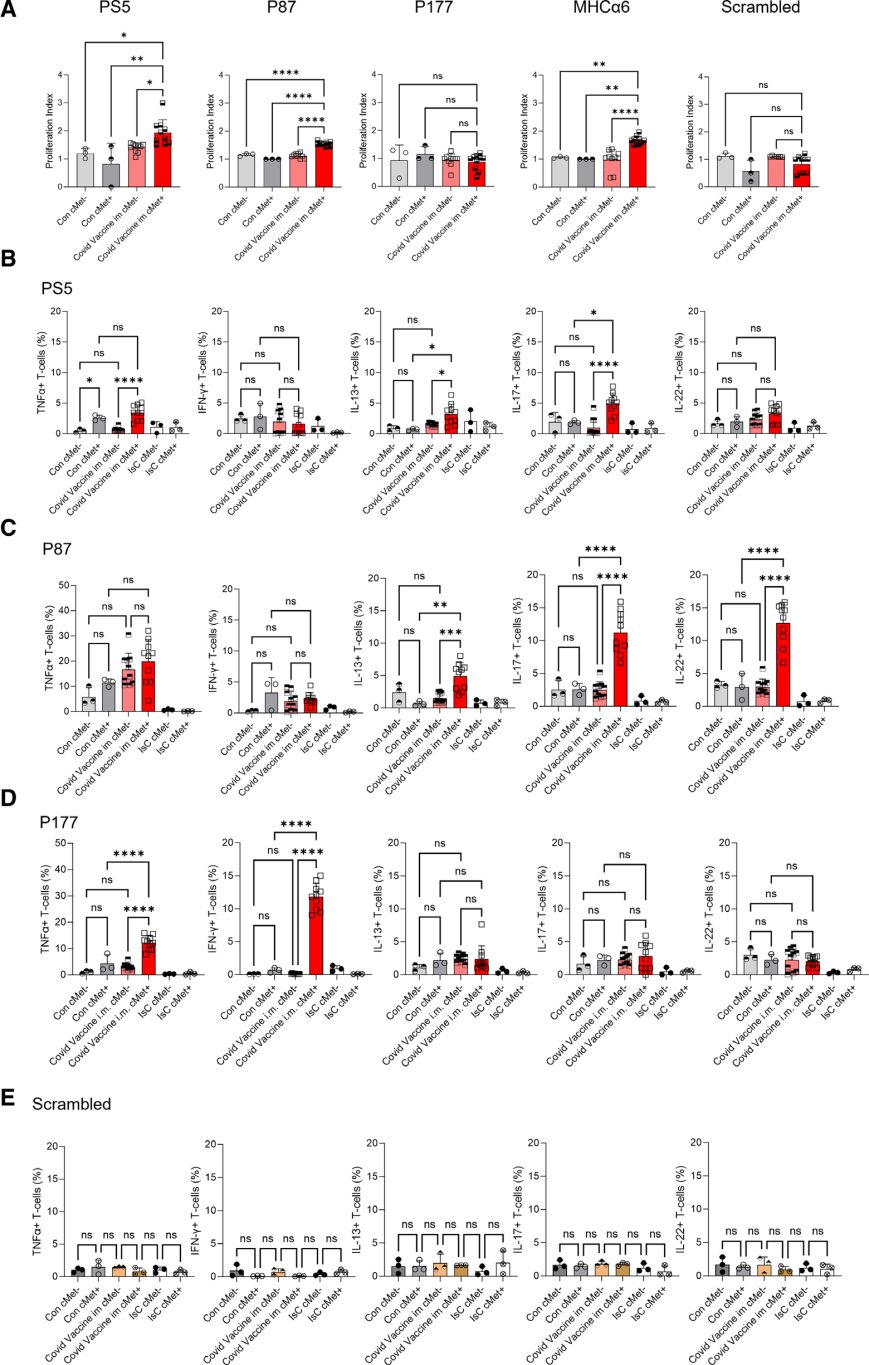
**Response of T cells from mRNA-1273-immunized mice to Spike epitopes.** T cells from mRNA-1273-immunized mice (day 21) were labeled with CFSE and rechallenged with the indicated peptides. Cells were harvested and analyzed 5 days later for CFSE dilution and cMet c-mesenchymal epithelial transition factor expression. The mean division index is shown in **A. B** through **E**, Production of the indicated cytokines by cMet^+^ and cMet^−^ T cells was measured 6 hours after restimulation with the indicated antigens by intracellular staining and flow cytometry. Statistical analysis was performed with 2-way ANOVA, followed by Tukey multiple comparison test. All data are shown as median±interquartile range. *P<0.05, **P<0.01, ***P<0.005, ****P<0.001 (N=3, control n=3, PS5 n=10, P87 n=10, P177 n=10).

As shown in Figure 2A and Figure S2A, cMet^+^ but not cMet^−^ memory T cells from mRNA-1273–vaccinated animals proliferated to PS5 and P87 but not P177. Additionally, cMet^+^ but not cMet^−^ T cells proliferated to the MHCα6 peptide, confirming their autoreactive nature.^35^ We further analyzed cytokine production by peptide-challenged T cells. In response to PS5, similar proportions of cMet^+^ and cMet^−^ CD4^+^ T cells produced TNFα (tumor necrosis factor α), whereas IL-13 and IL-17 were selectively produced by the cMet^+^ subset (Figure 2B; Figure S2B). IL-13 was used as a surrogate for Th2 responses, as IL-4 could not be measured by intracellular antibody staining. Rechallenge with P87 elicited the production of IL-13, IL-17, and IL-22 selectively by cMet^+^ T cells (Figure 2C). Despite the lack of proliferation, stimulation with P177 (Figure 2D) induced a significantly high proportion of TNFα- and IFN-γ (interferon γ)–producing cMet^+^ T cells.

### Human T-Cell Responses to SARS-CoV-2 Spike Protein–Derived Epitopes

We next sought to determine whether patients with Vacc-AMP had also developed an immune response against the epitopes described above by analyzing their peripheral blood mononuclear cells by flow cytometry (see the gating strategy in Figure S3). In addition, we assessed a cohort of patients who had been admitted to intensive care units because of severe COVID-19 (defined COVID-19) on the second day after admission.^44^ Of note, these patients did not present with cardiac symptoms or displayed severe sign of cardiac damage, as measured by BNP (B-type natriuretic peptide) and high-sensitivity cardiac troponin T values (Table S1). It was not possible to match these 2 cohorts for age and comorbidity for obvious reasons. Finally, we analyzed peripheral blood mononuclear cells from a group of individuals age and sex matched to the AMP cohort who had received the Moderna/Pfizer vaccine in their first and second vaccination but did not develop myocarditis (vaccinated healthy controls [Vacc-HC]). The Vacc-HC cohort received the RNA vaccine in the 12 months before blood sampling.

As shown in and Figure S4A and S4B (see gating strategy in Figure S3), there were comparable proportions of total memory CD4^+^ T cells and memory CD8^+^ T cells in the Vacc-HC, Vacc-AMP, and COVID-19 cohorts. Significantly increased levels of circulating cMet^+^ memory CD4^+^ and CD8^+^ T cells were detected in the COVID-19 and Vacc-AMP groups. Total and cMet^+^ Treg cell (Figure S4C) proportions were significantly higher in patients with COVID-19 compared with Vacc-AMP and Vacc-HC.

To ensure epitope recognition in the context of most human leukocyte antigen molecules, long-peptide epitopes (30-mer) were designed, containing the peptide sequences used in mice and defined by the addition of an h (human). Peptide sequences are provided in Supplemental Methods.

**Figure 3.**
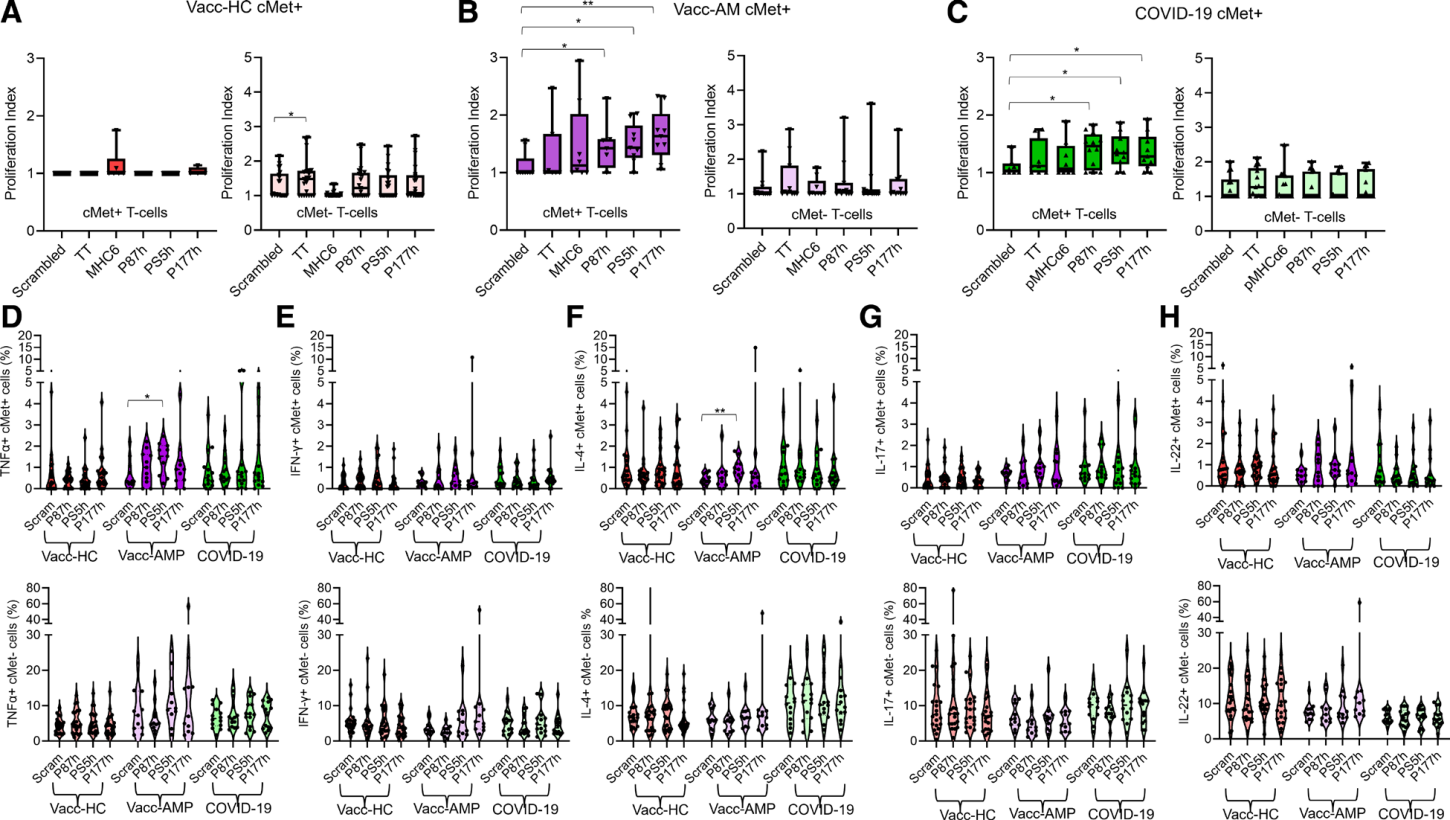
**Proliferation assays and cMet^+^ and cMet^−^ T-cell cytokine profiles after peptide stimulation.** PBMCs isolated from human blood were labeled with Tag-it-Violet and stimulated with 20 µg/mL of scrambled peptide; MHCα6 partial protein; P87h, PS5h, and P177h peptides; and tetanus toxoid (TT; 10 μg/mL) for 7 days. Proliferation assays show the proliferation index values of CD4^+^ cMet (c-mesenchymal epithelial transition factor)+, and cMet^−^ cells from peripheral blood mononuclear cells of vaccinated healthy controls (Vacc-HCs; **A**, n=21), patients with post-mRNA vaccination AMP (Vacc-AMP; **B**, n=11), and patients with COVID-19 (**C**, n=14). Statistical analysis was performed with Kruskal-Wallis test, followed by Dunn multiple comparisons test. The frequency of peripheral blood cMet^+^ and cMet− T cells producing TNFα (tumor necrosis factor α)^−^ (**D**), IFN-γ (interferon γ)^−^ (**E**), IL-4 (interleukin-4; **F**), IL-17^−^ (**G**), and IL-22^−^ (**H**) from the cohorts were analyzed after stimulation with the indicated peptides by intracellular staining and flow cytometry. Statistical analysis was performed with repeated-measures 1-way ANOVA with Dunnett multiple comparisons. All data are shown as median±interquartile range. *P<0.05, **P<0.01, ***P<0.001, ****P<0.0001.

Proliferation of cMet^−^ and cMet^+^ T cells to the Spike peptides, MHCα6 partial protein, a control scrambled peptide, and the recall antigen tetanus toxoid was analyzed by Tag-it Violet labeling and flow cytometry. As shown in Figure 3A through 3C and Figure S4D, cMet^+^ but not cMet^−^ CD4^+^ T cells from the COVID-19 and Vacc-AMP cohorts proliferated against Spike peptides P87h, PS5h, and P177h. Notably, we did not detect proliferation against the MHCα6 partial protein (amino acid 160-816) in any of the cohorts. CD4^+^ T cells from Vacc-HC individuals responded tetanus toxoid, and this response segregated to the cMet^−^ memory T cell subset.

We then sought to define the cytokine signature of peptide-responding CD4^+^ T cells. As shown in Figure 3D through 3H, cytokine production segregated to the cMet^+^ T cell subset. Compared with the other cohorts, the Vacc-AMP cohort displayed increased proportions of TNFα^+^ and IL-4^+^ T cells in response to PS5h. Of note, peptide-specific cytokine production by T cells from patients with COVID-19 could not be measured because of high responses to control scrambled peptide, in line with nonspecific T cell hyperactivation in severe COVID-19.^45^

Unlike in mice, no cytokine production was induced by P87h in any of the human cohorts.

Taken together, these data indicate that exposure to cross-reactive spike epitopes induces the differentiation of Th1/2 cardiotropic T cells, a signature partially shared with mRNA-1273 vaccine-induced AMP in mice (Figure 1) and viral/autoimmune myocarditis in humans.^35^ In patients with Vacc-AMP, upon stimulation with PS5h, these responses segregated in the cMet^+^ T-cell subset.

### The Human/Murine Shared Spike T-Cell Epitope PS5 Triggers Myopericarditis in Mice

We next sought to establish the ability of single Spike epitopes identified in mice and patients to elicit an antiheart autoimmune T-cell response in mice.

In a series of pilot experiments, different immunization routes and adjuvants were tested. The AS03 plus monophosphoryl lipid A adjuvant was chosen because of these pilot data. Intranasal immunization with a pool of the peptides and adjuvant resulted in the induction of T-cell recall responses ex vivo and the development of cardiac inflammatory infiltrates (not shown). In contrast, intramuscular or subcutaneous (footpad) immunization elicited recall proliferative responses but did not lead to the development of AMP. These data will be presented and discussed later (Figure S9).

Based on these observations, 5-week-old Balb/cAnN male mice were immunized with each peptide (0.6 mg/kg) intranasally and boosted with the same dose 14 days later. The dose was calculated based on pilot experiments. Control mice received adjuvant alone with the same schedule. Mice were euthanized 21 days after the first immunization.

**Figure 4.**
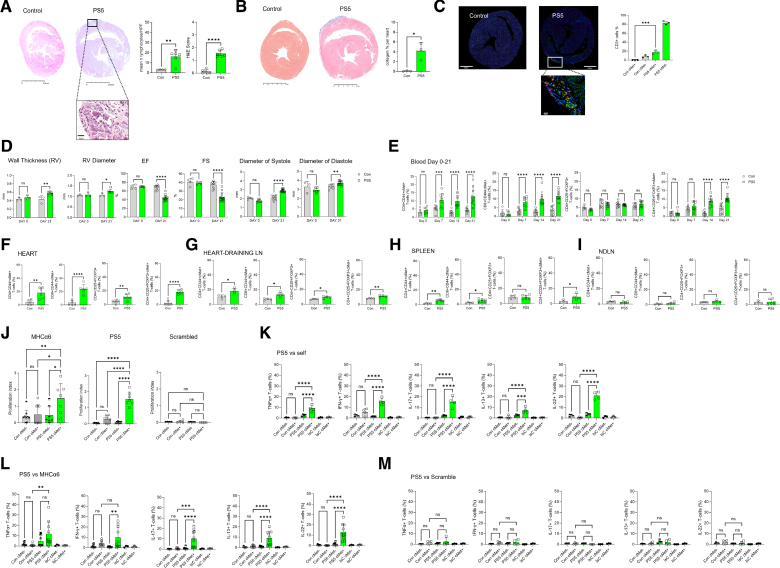
**Immunization with the spike peptide PS5 induces acute myopericarditis in susceptible mice.** Five-week-old male Balb/cAnN male mice were immunized with PS5 (0.6 mg/kg) in AS03 plus MPLA adjuvant intranasally and boosted with the same dose 14 days later. Control mice received adjuvant alone. The development of inflammatory infiltrates and collagen deposition were assessed by hematoxylin-eosin (**A**) and Masson trichrome staining (**B**) of the heart 21 days after the first immunization. Column graphs show lymphocyte infiltration and disease scores (**A**) obtained as described in Methods. Representative images at 10× and 40× magnification are shown on the **right** (scale bar=2.5 mm and 50 µm, respectively). Statistical analysis was performed with unpaired Student *t* test. **C**, Immunofluorescence staining with the indicated markers was performed on heart tissue harvested on day 21. The bar graph shows the mean percentage of cMet (c-mesenchymal epithelial transition factor)^+^ and cMet^−^ T cells measured by QuPath. Statistical analysis was performed with 2-way ANOVA, followed by Tukey multiple comparisons test. **D**, Right ventricle (RV) wall thickness, RV diameter, left ventricle ejection fraction (EF), fractional shortening (FS), and diameter at systole and diastole were measured by echocardiography at baseline (day 0) and 21 days after the first immunization. Statistical analysis was performed with 2-way mixed-effects ANOVA, followed by Tukey multiple comparisons test. **E**, Tail vein blood was sampled on the same mouse at the indicated time points. The percentage of the indicated T-cell populations was determined by flow cytometry. Statistical analysis was performed with 2-way mixed-effects ANOVA, followed by Tukey multiple comparison test. **F** through **I**, Percentage of cMet^+^ T cells measured in single-cell suspensions from whole hearts and heart-draining and nondraining lymph nodes. Statistical analysis was performed with unpaired Student *t* test. **J**, T cells from PS5-immunized mice (day 21) were labeled with CFSE and rechallenged with the indicated peptides. Cells were harvested and analyzed 5 days later for CFSE dilution and cMet expression. The mean division index is shown. Statistical analysis was performed with 2-way ANOVA, followed by Tukey multiple comparisons test. **K** through **M**, Production of the indicated cytokines by cMet^+^ and cMet^−^ T-cells was measured 6 hours after restimulation with the indicated antigens by intracellular staining and flow cytometry. Statistical analysis was performed with 2-way ANOVA, followed by Tukey multiple comparison test. All data are represented as median±interquartile range. *P<0.05, **P<0.01, ***P<0.005, ****P<0.001 (N=3, control n=3, PS5 n=10, P87 n=10, P177 n=10).

Immunization of mice with peptides PS5 (Figure 4) and P87 (Figure S5), but not P177 (Figure S6), led to the development of cardiac inflammatory injuries in the pericardium and myocardium (Figure 4A through 4C; Figure S7A through S7C) and raised cardiac troponin I levels in PS5-immunized animals (Figure S7D), similar to those we observed after mRNA vaccination. Immunofluorescence analysis identified numerous cMet^+^ T cells in these inflammatory infiltrates. Echocardiography detected functional alterations, including in the right ventricle, consistent with AMP (Figure 4D).

Immunization with PS5 was accompanied by a progressive increase in circulating CD44^+^ cMet^+^ CD4^+^ and CD8^+^ T cells and Treg cells (Figure 4E). Increased percentages of both CD44^+^ cMet^+^ CD4^+^, and CD8^+^ T cells and Treg cells were detected in the heart, heart dLNs, and the spleen but not non-dLNs of immunized mice euthanized on day 21 (Figure 4F through 4I). Proliferative responses were detected upon rechallenge with PS5, and MHCα6, suggesting that self-reactive antimyocardial T cells can be triggered by immune responses to SARS-Cov-2 antigens, but not with a scrambled peptide (Figure 4J).

A higher proportion of CD4^+^ T cells from immunized mice but not controls produced TNFα, IFN-γ, IL-17, IL-13, and IL-22 in response to a rechallenge with PS5 and MHCα6, and this response segregated to the cMet^+^ T-cell subset (Figure 4K through 4M).

Immunization with P87 led to pathology and T-cell functional responses similar to those observed in PS5-immunized mice, with the exception that IL-22 was not produced in response to a rechallenge with the immunizing peptide (Figure S5).

### T-Cell Responses to the Kv2.1 Autologous Peptide in Patients with Vacc-AMP and COVID-19 and in PS5-Immunized Mice

Given that PS5 elicited a strong proliferative response and cytokine production in patients with Vacc-AMP, and that this peptide could induce AMP in mice, we focused our subsequent investigations on this epitope. To establish the occurrence of molecular mimicry in the context of exposure to spike immunization, we sought to test the proliferation and functional phenotype of T cells from mice previously immunized intranasally with PS5 and from the patient cohorts upon challenge with the autologous, Kv2-derived peptide.

**Figure 5.**
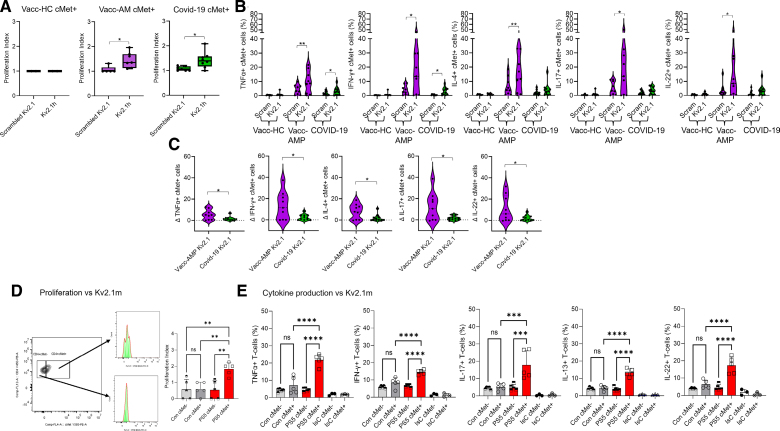
**Patients with VACC-AMP and PS5-immunized mice display functional T-cell responses to the homologous Kv2 peptide.** PBMCs isolated from human blood were labeled with Tag-it-Violet and stimulated with 200 ng/mL scrambled and autologous Kv2.1h (human) peptides for 7 days. **A**, Mean proliferation index of CD4^+^ cMet (c-mesenchymal epithelial transition factor)^+^ T cells from peripheral blood mononuclear cells (PBMCs) of vaccinated healthy controls (Vacc-HC; n=8), patients with post-mRNA vaccination AMP (Vacc-AMP; n=9), and patients with COVID-19 (n=10) patients measured by flow cytometry. Statistical analysis was performed with paired Student *t* test. **B**, The frequency of cytokine-producing peripheral blood cMet^+^ T cells among peripheral blood cMet^+^ T cells from the cohorts were analyzed after stimulation with the indicated peptides, using intracellular staining and flow cytometry. Statistical analysis was performed with paired Student *t* test. **B**, Frequencies of cytokine-producing cMet^+^ T cells in response to the scrambled peptide were subtracted from those of cMet^+^ T cells stimulated with Kv2.1 peptide in a paired manner. Statistical analysis was performed with paired Student *t* test. **C**, To compare the size of cytokine-producing T cell subsets in patients with Vacc-AMP (n=9) and COVID-19 (n=10), frequencies of cytokine producers in response to scrambled peptide were subtracted in a paired manner from those in response to the Kv2.1h peptide. Statistical analysis was performed with unpaired Student *t* test. **D**, Five-week-old Balb/cAnN male mice were immunized with PS5 (0.6 mg/kg) in AS03 plus MPLA adjuvant intranasally and boosted with the same dose 14 days later. Control mice received adjuvant alone. T cells from PS5-immunized mice (day 21) were labeled with CFSE and rechallenged with the homologous Kv2.1m (murine) peptide. Cells were harvested and analyzed 5 days later for CFSE dilution and cMet expression by flow cytometry. The mean division index of cMet^−^ and cMet^+^ T cells from controls and immunized mice is shown. Statistical analysis was performed with 2-way ANOVA, followed by Tukey multiple comparison test. **E**, Production of the indicated cytokines by cMet^+^ and cMet^−^ T-cells was measured 6 hours after restimulation with the Kv2.1m peptide by intracellular staining and flow cytometry. Statistical analysis was performed with 2-way ANOVA, followed by Tukey multiple comparison test. Data are shown as median±interquartile range (**A** through **C**) or median±interquartile range (**D**). *P<0.05, **P<0.01, ***P<0.005, ****P<0.001 (N=3, control n=5, PS5 n=5).

CD4^+^ cMet^+^ T cells from patients with Vacc-AMP and COVID-19 displayed a significant proliferative response to the Kv2.1 peptide (Figure 5A; Figure S8A). A high proportion of Vacc-AMP cMet^+^ but not cMet^−^ T cells produced TNFα and IL-4 upon PS5h challenge but also IFN-γ, IL-17, and IL-22 in response to the Kv2.1 human peptide (Figure 5B; Figure S8B), similar to what we observed in mRNA-1273-immunized mice. Despite similar levels of proliferation, the cytokine response by T cells from the COVID-19 cohort was significantly blunted compared with that of Vacc-AMP T cells (Figure 5C).

To validate these finding in the murine AMP model, male Balb/cAnN mice were immunized intranasally with the PS5 peptide (0.6 mg/kg) in adjuvant and boosted with the same dose 14 days later. Control mice received adjuvant alone with the same schedule. As shown in Figure 5D and 5E, T cells from immunized mice, which also developed myocarditis, proliferated against the murine Kv2 epitope and produced a cytokine response very similar to the one observed in patients. Control mice did not display T-cell activation, suggesting that the response was instigated by previous immunization with PS5; ie, mediated by memory T cells rather than arising from a distinct naïve T-cell response.

### Acquisition of Cardiotropism (cMet Expression) Is Necessary for the Development of Myocarditis Upon Immunization With Spike Epitopes

The evidence that most of the measured immune responses to Spike epitopes were segregated to the cardiotropic cMet^+^ T-cell subset suggests that the acquisition of cardiac homing is necessary to elicit a cardiac inflammatory response. Systemic antigen distribution occurs after RNA vaccination, and RNA vaccines have inherent adjuvanticity, enabling them to also elicit a systemic inflammatory response.^21^ Although administered peptides can also disseminate systemically, their ability to induce immunization is dependent on adjuvant-induced local inflammation. As previously mentioned, only intranasal immunization with PS5 led to the development of AMP in mice, whereas intramuscular immunization in the forelimb (as in the mRNA vaccine) or subcutaneous immunization in the footpad (a site not sharing lymph nodes with the heart) elicited recall proliferative responses selectively by cMet^−^ cells but did not lead to the development of AMP (Figure S9). Further, there was no increase of cMet^+^ T cells in these mice, and the recall responses in the cMet^−^ subset were associated with TNFα and IFN-γ production. Crucially, the heart and lungs share mediastinal dLNs,^46,47^ which are sites of priming permissive of the acquisition of cardiotropism.

We have previously shown that pharmacological inhibition of cMet at the time of homing imprinting can prevent the development of cardiotropic T cells and myosin-induced autoimmune myocarditis.^34,35^

Male Balb/cAnN mice were immunized intranasally with the PS5 peptide (0.6 mg/kg) and boosted with the same dose 14 days later. Control mice received adjuvant alone with the same schedule. A third group of mice received the cMet-selective small molecule inhibitor PHA-665752 (INH, 9.6 mg/kg per day) for 10 days starting on day 7 after the initial immunization, a time frame when topographical memory is thought to occur.^34^ Mice were euthanized 21 days after the first immunization.

**Figure 6.**
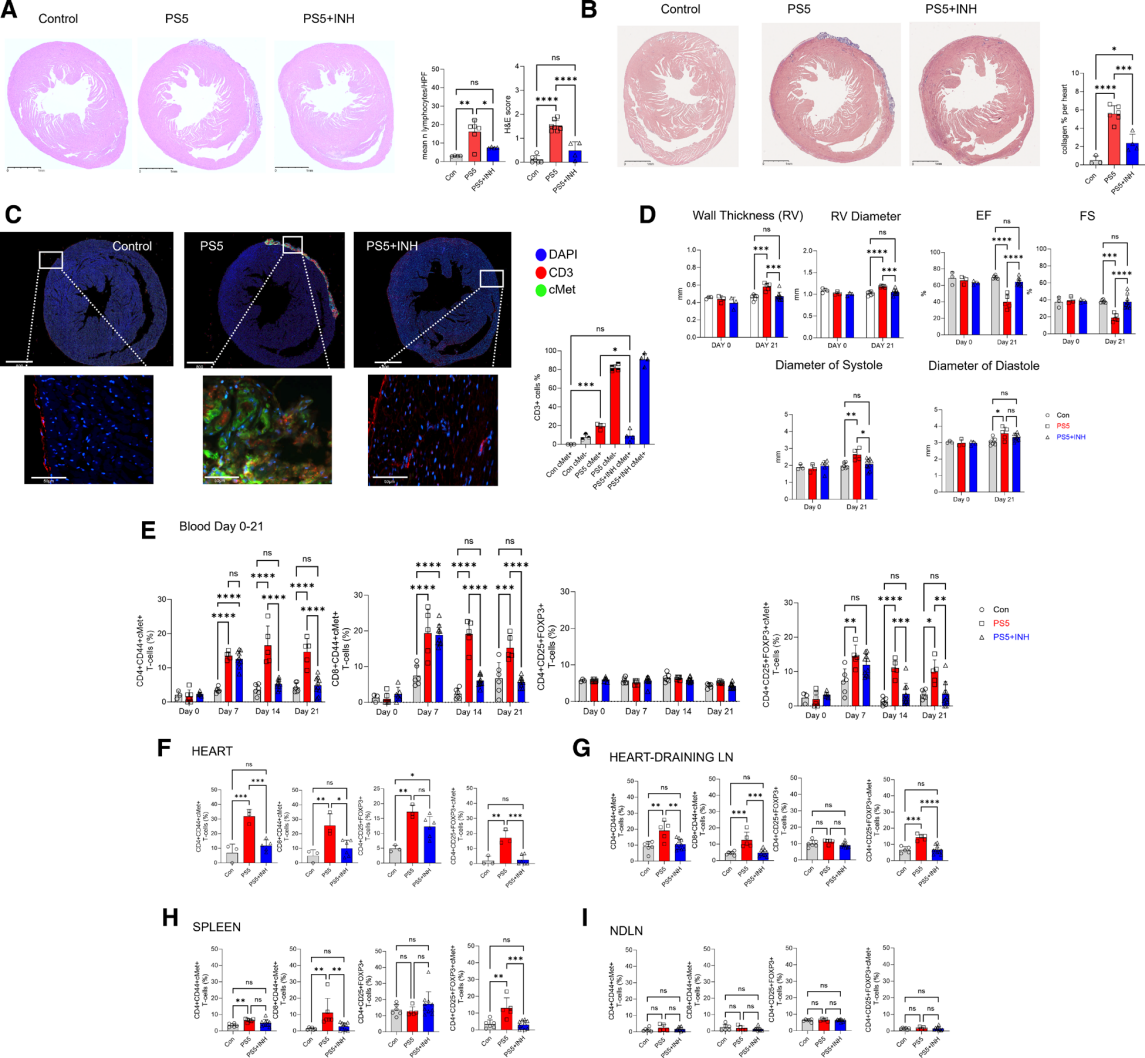
**Pharmacological cMet inhibition substantially reduces the severity of PS5-induced myopericarditis in mice.** Five-week-old Balb/cAnN mice were immunized with PS5 (0.6 mg/kg) in AS03 plus MPLA adjuvant intranasally and boosted with the same dose 14 days later. Control mice received adjuvant alone. Some mice received intraperitoneal injections of the cMet (c-mesenchymal epithelial transition factor) inhibitor PHA-665752 (9.6 mg/kg, PS5+INH) from day 8 to day 17 after immunization. The development of inflammatory infiltrates and collagen deposition were assessed by hematoxylin-eosin (**A**) and Masson trichrome staining (**B**) of the heart 21 days after the first immunization. Column graphs show lymphocyte infiltration and disease scores (**A**) obtained as described in Methods. Representative images at 10× and 40× magnification are shown on the **right** (scale bar=2.5 mm). Statistical analysis was performed with 1-way ANOVA, followed by Dunnett multiple comparison. **C**, Immunofluorescence staining with the indicated markers was performed on heart tissue harvested on day 21. The bar graph shows the mean percentage of cMet^+^ and cMet^−^ T cells measured by QuPath. Statistical analysis was performed with 2-way ANOVA, followed by Tukey’s multiple comparisons test. **D**, Right ventricle (RV) wall thickness, RV diameter, left ventricle ejection fraction (EF), fractional shortening (FS), and diameter at systole and diastole were measured by echocardiography at baseline (day 0) and 21 days after the first immunization. Statistical analysis was performed with 2-way mixed-effects ANOVA, followed by Tukey multiple comparison test. **E**, Tail vein blood was sampled on the same mouse at the indicated time points. The percentage of the indicated T cell populations was determined by flow cytometry. Statistical analysis was performed with 2-way mixed-effects ANOVA, followed by Tukey multiple comparison test. **F** through **I**, Percentage of cMet^+^ T cells measured in single-cell suspensions from whole hearts, heart-draining lymph nodes, spleen, and nondraining lymph nodes. Statistical analysis was performed with 1-way ANOVA, followed by Dunnett multiple comparison. All data are shown as median±interquartile range. *P<0.05, **P<0.01, ***P<0.005, ****P<0.001 (N=3, control n=6, PS5 n=5, PS5+INH n=10).

Immunization of mice with PS5 peptide led to AMP development on the basis of the presence and size of mononuclear cell infiltrates and collagen deposition in the myocardium (Figure 6A and 6B). A large proportion of infiltrating CD3^+^ T cells were cMet^+^ (Figure 6C). Echocardiography revealed functional alterations consistent with AMP (Figure 6D). Importantly, pharmacological inhibition of cMet (PS5+INH) significantly reduced experimental autoimmune myocarditis incidence, mononuclear cell infiltrates, collagen deposition, and cMet^+^ T cell infiltration as well as the echocardiographic correlates of AMP.

The proportion of circulating memory CD44^+^cMet^+^ T cells was monitored by flow cytometry for the duration of the experiment. Development of disease was marked by a progressive increase of circulating CD44^+^ cMet^+^ T cells in immunized mice (Figure 6E). Conversely, in mice treated with the cMet inhibitor, reduction of disease severity was mirrored by a decrease in circulating cMet^+^ T cells.

We further analyzed the distribution of cMet^+^ T-cells ex vivo on day 21. Increased proportions of both CD4^+^ and CD8^+^ cMet^+^ memory T cells were found in heart tissue, heart dLNs, and the spleen but not in non-dLN (Figure 6F through 6I). In addition, we detected a significant increase in cMet^+^CD4^+^CD25^high^FoxP3^+^ Treg cells in the heart, dLNs, and spleen but not in non-dLNs. Treatment with PHA-665752 blunted the expansion of cMet^+^ T cells in the heart, dLNs, and spleen and reduced the proportion of Treg cells.

**Figure 7.**
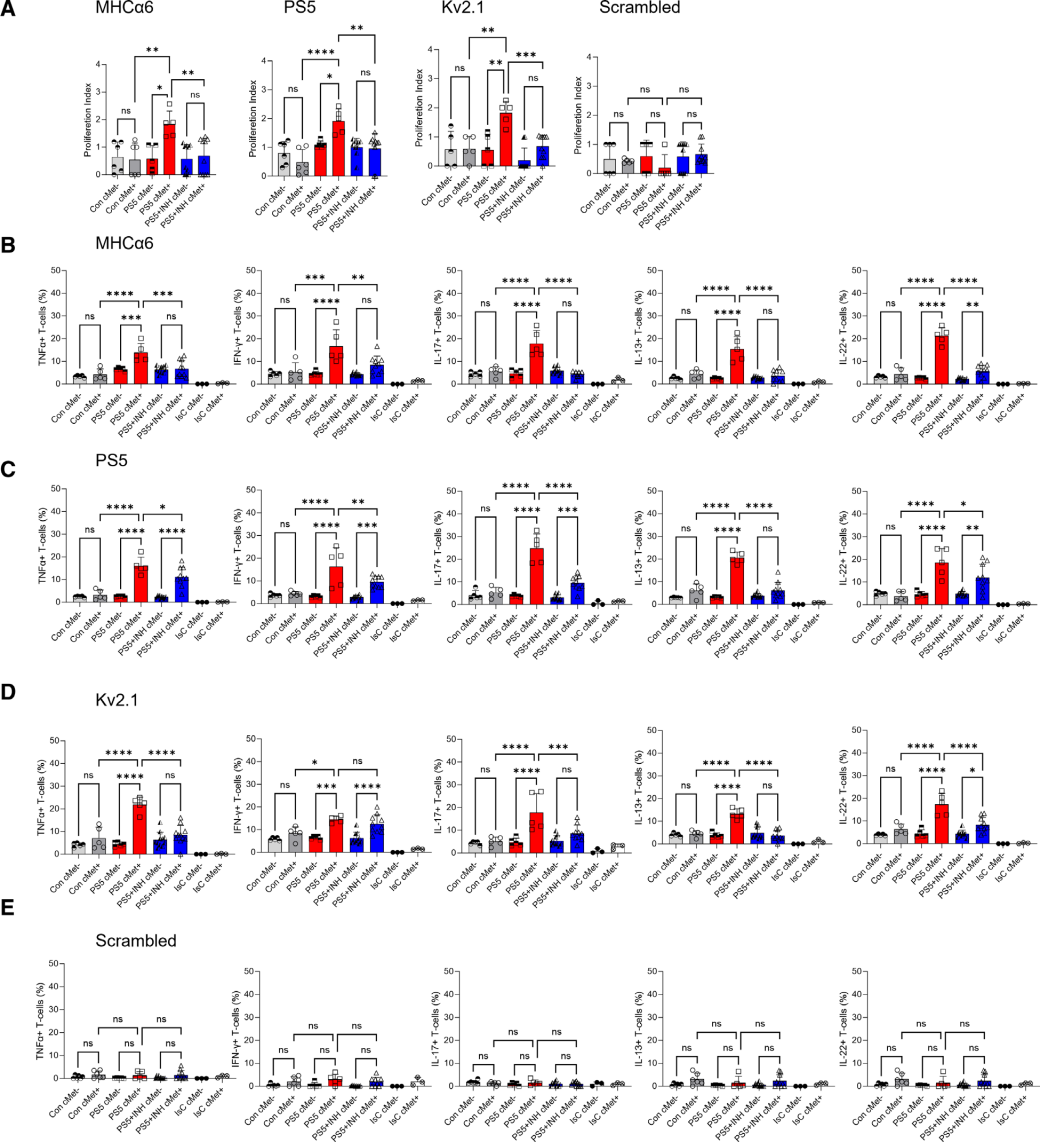
**Pharmacological cMet inhibition reduces functional responses by cMet^+^ T cells in PS5-immunized mice.** T cells from PS5-immunized mice (day 21) were labeled with CFSE and rechallenged with the indicated peptides. Cells were harvested and analyzed 5 days later for CFSE dilution and cMet (c-mesenchymal epithelial transition factor) expression. **A**, Mean division index of cMet^−^ and cMet^+^ T cells from controls and immunized mice with and without cMet inhibitor. Statistical analysis was performed with 2-way ANOVA, followed by Tukey multiple comparison test. **B** through **E**, Production of the indicated cytokines by cMet^+^ and cMet^−^ T cells was measured 6 hours after restimulation with the indicated antigens by intracellular staining and flow cytometry. Statistical analysis was performed with 2-way ANOVA, followed by Tukey multiple comparison test. All data are shown as median±interquartile range. *P<0.05, **P<0.01, ***P<0.005, ****P<0.001 (N=3, control n=6, PS5 n=5, PS5+INH n=10).

Proliferation of T cells from immunized mice to cardiac myosin, PS5, and the homologous peptide Kv2.1 was blunted upon treatment with the cMet inhibitor (Figure 7A). Similarly, pharmacological blockade of cMet significantly reduced the proportion of cMet^+^ T cells producing TNFα, IFN-γ, IL-17, IL-13, and IL-22 (Figure 7B through 7E).

Taken together, these data suggest that the acquisition of cardiotropism is essential for the development of AMP in mice.

## Discussion

In this study, we identified an RNA vaccine–encoded Spike epitope with homology to a cardiac self-peptide mapping to the voltage-dependent K+ channel Kv2 (PS5), which elicits strong functional T-cell responses in patients with post-RNA vaccine AMP and induces AMP in susceptible mice upon intranasal immunization, with striking similarities to human disease.^32^

Three peptide epitopes were selected and tested on the basis of their homology with peptide sequences in cardiac proteins and a known ability to induce T-cell responses against SARS-Cov-2 in mice. Of these, we propose that mimicry with PS5 is the most likely or frequent inducer of cardiac autoimmunity for the following reasons. First, PS5 induced recall responses in both vaccinated patients and mice. Second, the cytokine response against the autologous Kv2 epitope in the AMP-Vacc cohort expands to include IL-17, IFN-γ, and IL-22, similar to that of autoimmune T cell responses in human and experimental myocarditis.^35,48,49^ Finally, immunization with PS5 led to the development of AMP in mice. Homolog epitopes capable of reproducing the corresponding human disease in mice have been identified for several autoimmune diseases,^50–52^ including myocarditis.^53^

Molecular mimicry is an antigen-specific mechanism of autoimmunity that occurs when similarities between pathogen and self-peptides induce activation of autoreactive T cells in a susceptible individual. T cells from patients with Vacc-AMP and COVID-19 proliferated to the autologous Kv2-derived peptide at similar levels. However, the proportion of cytokine-producing T cells was significantly higher in Vacc-AMP, suggesting uncoupled proliferation and cytokine production in T lymphocytes from patients with COVID-19. The ability of T cells to proliferate without necessarily producing large amounts of cytokines can be influenced by the affinity of the T-cell receptor (TCR) for its antigen, with weaker TCR affinities often leading to greater infidelity between proliferation and robust cytokine production.^54^ Moreover, a recent clinical trial showed that T cells engineered to express an affinity-enhanced TCR directed to an epitope of the melanoma MAGE-A3 are also able to effectively recognize a similar peptide epitope derived from the entirely unrelated cardiac protein titin, resulting in severe cardiac toxicity.^55^

Therefore, molecular mimicry per se is unlikely to be the only underlying mechanism for the initiation of autoimmune responses; other factors, such as host genetics and the affinity of the TCR repertoire, determine the occurrence of autoimmunity. This is reflected by the absence of immune-mediated cardiac injury in our COVID-19 cohort despite these patients having severe systemic inflammation and displaying raised circulating cardiotropic T cells that proliferated in response to some of the Spike epitopes.

Of note, the proportions of circulating Treg cells from patients with COVID-19 were significantly higher when compared with those measured in the Vacc-AMP cohort, suggesting potential differences in regulatory mechanisms.

T cells from individuals who received the mRNA vaccines without developing cardiac side effects, including AMP, did not display immune responses to the same epitopes, indicating an abortive T-cell response, possibly because of the progressive loss of T-cell memory towards Spike after vaccination.

We show that the ability of T cells to migrate to the heart is instrumental for the development of cardiac autoimmunity. The acquisition of tissue-specific homing requires the delivery, during T-cell priming, of additional signals by dendritic cells, which pick up tissue-specific cues and transport them to the tissue dLNs. These additional signals promote the expression of a specialized set of homing receptors by primed T cells, which facilitate their migration to target tissue. The HGF/cMet axis plays a major role in imprinting T-cell cardiotropism, and the localization of antigen to lymph nodes draining the heart is crucial for this effect.^35^

The systemic biodistribution of RNA vaccines and of their products in human tissues^20,22–24^ and their intrinsic adjuvant activity^21^ are crucial for this event. Because of their systemic availability, mRNA vaccines are more likely to reach lymph nodes draining multiple tissues and induce systemic inflammation via IL-1.^21^ In contrast, the nontoxic LPS derivative monophosphoryl lipid A, a component of the adjuvant we used in this study and that promotes inflammasome activation and IL-1 production^56^ selectively induced T-cell cardiotropism when administered in sites that share lymph nodes with the heart and a source of HGF (lungs, intranasal route)^57,58^ but not in other sites not meeting these conditions (footpad subcutaneous tissue and forepaw muscle).

In this context, we also found increased numbers of cMet^+^ T cells in the COVID-19 cohort, which did not develop myocarditis, likely because of HGF production by inflamed lung tissue. Because this group of patients is epidemiologically very different from the AMP cohort, we suggest that additional important information can be drawn from this comparison. Despite similar immunoreactivity and the presence of high levels of cMet^+^ T cells in the patients with COVID-19, these did not develop AMP. This indicates that genetic susceptibility is a key determinant of AMP development,^59,60^ like in the murine model. Although sex and age bias in myocarditis has been ascribed to the immune-enhancing effect of testosterone,^61^ the emerging association of this condition with gene variants encoding for cardiac structural proteins^62^ and specific human leukocyte antigen haplotypes^63^ might also explain susceptibility to post-vaccine AMP.

Potential side effects of future mRNA vaccines, or any vaccine with systemic distribution, might therefore be related to their ability to generate cohorts of T cells endowed with a range of organ-specific tropisms.

Accordingly, we speculate that molecular mimicry coincident with the acquisition of variable or multiple-organ homing potential as a result of systemic biodistribution (viremia or mRNA vaccination) might contribute to the pathogenesis of these conditions. Although we did not analyze antibodies to Spike protein in our patients or mRNA in cardiac tissue samples, others have found mRNA in the human heart and non-neutralizing antibodies and circulating Spike protein in individuals with vaccine-related myocarditis.^59,64^

We recognize that the limited information available from our small human cohorts does not permit further interrogation of this hypothesis. In addition, only male mice were used to reflect epidemiology, hence information on the immunoreactivity and disease susceptibility in female mice is not included. However, the evaluation of RNA vaccines for their potential to induce T cells with tropism for multiple organs may help avoid side effects or guide more appropriately targeted immunization strategies.

Undoubtedly, although the potency of mRNA vaccination has unleashed the potential to swiftly overcome global lethal infections and saved countless lives in the SARS-Cov-2 pandemic, we propose the paradigm “mimicry, TCR affinity and, importantly, homing imprinting,” which should help refine this invaluable strategy for immune protection against existing and emerging pathogens.

## Article Information

### Acknowledgments

The authors would like to thank all patients and healthy blood donors whose generosity allowed us to conduct this study. This work forms part of the research areas contributing to the translational research portfolio of the Cardiovascular Biomedical Research Unit at Barts, which is supported and funded by the National Institute for Health Research.

### Author Contributions

Dr Fanti, formal analysis and investigation; Dr Dyer, formal analysis and investigation; Dr Ingimarsdóttir, conceptualization, formal analysis, and funding acquisition; Dr Harding, funding acquisition and resources; Dr Wang, investigation; Dr D’Amati, formal analysis; Dr Shahaj, conceptualization; Dr Sigurbergsdóttir, resources; Dr Thórsdóttir, resources; Dr Gunnarsdóttir, resources; Dr Kanoni, formal analysis; Dr Wright, resources; Dr Martin, resources; Dr Chorlton, resources; Dr Hollowood, resources; Dr Brynjólfsson, resources; Dr Lúdvíksson, resources; Dr Bert, resources; Dr Keane, resources; Dr Mohiddin, conceptualization and resources; Dr Longhi, conceptualization, funding acquisition, and supervision; Dr Marelli-Berg, conceptualization, funding acquisition, and supervision.

### Sources of Funding

This work was supported by the British Heart Foundation (FS/CRTF/20/24058 to Drs Marelli-Berg, Harding, and Mohiddin; RG/20/8/34995 to Drs Morelli-Berg and Longhi; CH/15/2/32064 and AA/18/5/34222 to Dr Morelli-Berg; and FS/4yPhD/F/22/34174B to Drs Keane and Longhi), the Barts Charity (MGU0413 to Dr Longhi), and the Landspítali Science Fund, Iceland (A-2022-21 and A-2024-27 to Dr Ingimarsdóttir) and donations from the Thompson, Hughes, and Parsons Foundations (to Dr Marelli-Berg).

### Disclosures

Dr Marelli-Berg is a consultant for AstraZeneca.

### Supplemental Material

Supplemental Methods

Tables S1–S3

Figure S1–S9

References 51–64

## Supplementary Material
