# Supplementary material for: Combined Adaptive Immune Mechanisms Mediate Cardiac Injury After COVID-19 Vaccination
Source: Circulation. 2025 Oct 30;152(21):1485–500. doi: 10.1161/CIRCULATIONAHA.125.074644 (PMC12643570; doi:10.1161/CIRCULATIONAHA.125.074644)
Supplement: Supplementary file 1 [file cir-152-1485-s001.docx]

# Reporting checklist for study using laboratory animals.

Based on the ARRIVE guidelines.

## Instructions to authors

Complete this checklist by entering the page numbers from your manuscript where readers will find each of the items listed below.

Your article may not currently address all the items on the checklist. Please modify your text to include the missing information. If you are certain that an item does not apply, please write "n/a" and provide a short explanation.

Upload your completed checklist as an extra file when you submit to a journal.

In your methods section, say that you used the ARRIVEreporting guidelines, and cite them as:

Percie du Sert N, Hurst V, Ahluwalia A, Alam S, Avey MT, Baker M, Browne WJ, Clark A, Cuthill IC, Dirnagl U, Emerson M, Garner P, Holgate ST, Howells DW, Karp NA, Lazic SE, Lidster K, MacCallum CJ, Macleod M, Pearl EJ, Petersen O, Rawle F, Peynolds P, Rooney K, Sena ES, Silberberg SD, Steckler T and Wurbel H. The ARRIVE Guidelines 2.0: updated guidelines for reporting animal research.

|  |  | Reporting Item | Page Number |
| --- | --- | --- | --- |
| **Essential 10** |  |  |  |
| Study design | [#1a](https://www.goodreports.org/reporting-checklists/arrive2/info/#1a) | Give details of the groups being compared, including control groups. If no control group has been used, the rationale should be stated. | All figure legends |
| Study design | [#1b](https://www.goodreports.org/reporting-checklists/arrive2/info/#1b) | Give details of the experimental unit (e.g., a single animal, litter, or cage of animals). | All figure legends |
| Sample size | [#2a](https://www.goodreports.org/reporting-checklists/arrive2/info/#2a) | Specify the exact number of experimental units allocated to each group, and the total number in each experiment. Also indicate the total number of animals used. | All figure legends |
| Sample size | [#2b](https://www.goodreports.org/reporting-checklists/arrive2/info/#2b) | Explain how the sample size was decided. Provide details of any a priori sample size calculation, if done. | Supplemental Material page 2-3 |
| Inclusion and exclusion criteria | [#3a](https://www.goodreports.org/reporting-checklists/arrive2/info/#3a) | Describe any criteria used for including or excluding animals (or experimental units) during the experiment, and data points during the analysis. Specify if these criteria were established a priori. If no criteria were set, state this explicitly. | Supplemental Material page 2-3 |
| Inclusion and exclusion criteria | [#3b](https://www.goodreports.org/reporting-checklists/arrive2/info/#3b) | For each experimental group, report any animals, experimental units, or data points not included in the analysis and explain why. If there were no exclusions, state so. | Supplemental Material page 2-3 |
| Inclusion and exclusion criteria | [#3c](https://www.goodreports.org/reporting-checklists/arrive2/info/#3c) | For each analysis, report the exact value of n in each experimental group. | All figure legends |
| Randomisation | [#4a](https://www.goodreports.org/reporting-checklists/arrive2/info/#4a) | State whether randomisation was used to allocate experimental units to control and treatment groups. If done, provide the method used to generate the randomisation sequence. | Supplemental Material page 2-3 |
| Randomisation | [#4b](https://www.goodreports.org/reporting-checklists/arrive2/info/#4b) | Describe the strategy used to minimise potential confounders such as the order of treatments and measurements, or animal/cage location. If confounders were not controlled, state this explicitly. | Supplemental Material page 2-3 |
| Blinding | [#5](https://www.goodreports.org/reporting-checklists/arrive2/info/#5) | Describe who was aware of the group allocation at the different stages of the experiment (during the allocation, the conduct of the experiment, the outcome assessment, and the data analysis). | Supplemental Material page 2-3 |
| Outcome measures | [#6a](https://www.goodreports.org/reporting-checklists/arrive2/info/#6a) | Clearly define all outcome measures assessed (e.g., cell death, molecular markers, or behavioural changes). | Supplemental Material page 2-3 |
| Outcome measures | [#6b](https://www.goodreports.org/reporting-checklists/arrive2/info/#6b) | For hypothesis-testing studies, specify the primary outcome measure, i.e., the outcome measure that was used to determine the sample size. | Supplemental Material page 2-3 |
| Statistical methods | [#7a](https://www.goodreports.org/reporting-checklists/arrive2/info/#7a) | Provide details of the statistical methods used for each analysis, including software used. | Figure legends,  Page 8-9 |
| Statistical methods | [#7b](https://www.goodreports.org/reporting-checklists/arrive2/info/#7b) | Describe any methods used to assess whether the data met the assumptions of the statistical approach, and what was done if the assumptions were not met. | Page 8-9 |
| Experimental animals | [#8a](https://www.goodreports.org/reporting-checklists/arrive2/info/#8a) | Provide species-appropriate details of the animals used, including species, strain and substrain, sex, age or developmental stage, and, if relevant, weight. | Figure legends,  Page 8 |
| Experimental animals | [#8b](https://www.goodreports.org/reporting-checklists/arrive2/info/#8b) | Provide further relevant information on the provenance of animals, health/immune status, genetic modification status, genotype, and any previous procedures. | Figure legends,  Page 8 |
| Experimental procedures | [#9a](https://www.goodreports.org/reporting-checklists/arrive2/info/#9a) | For each experimental group, including controls, describe the procedures in enough detail to allow others to replicate what was done, how it was done, and what was used. | Figure legends,  Supplemental Material page 2-3 |
| Experimental procedures | [#9b](https://www.goodreports.org/reporting-checklists/arrive2/info/#9b) | Timing and frequency of procedures | Figure legends |
| Experimental procedures | [#9c](https://www.goodreports.org/reporting-checklists/arrive2/info/#9c) | Where procedures were carried out (including detail of any acclimatisation periods). | Supplemental Material page 2-3 |
| Experimental procedures | [#9d](https://www.goodreports.org/reporting-checklists/arrive2/info/#9d) | Rationale for procedures | Supplemental Material page 2-3 |
| Results | [#10a](https://www.goodreports.org/reporting-checklists/arrive2/info/#10a) | For each experiment conducted, including independent replications, report summary/descriptive statistics for each experimental group, with a measure of variability where applicable (e.g., mean and SD, or median and range). |  |
| Figure legends |  |  |  |
| Results | [#10b](https://www.goodreports.org/reporting-checklists/arrive2/info/#10b) | If applicable, for each experiment conducted, including independent replications, report the effect size with a confidence interval. | Figure legends |
| **Recommended set** |  |  |  |
| Abstract | [#11](https://www.goodreports.org/reporting-checklists/arrive2/info/#11) | Provide an accurate summary of the research objectives, animal species, strain and sex, key methods, principal findings, and study conclusions. | Page 3 |
| Background | [#12a](https://www.goodreports.org/reporting-checklists/arrive2/info/#12a) | Include sufficient scientific background to understand the rationale and context for the study, and explain the experimental approach. | Page 6-7 |
| Background | [#12b](https://www.goodreports.org/reporting-checklists/arrive2/info/#12b) | Explain how the animal species and model used address the scientific objectives and, where appropriate, the relevance to human biology. | Page 10 |
| Objectives | [#13](https://www.goodreports.org/reporting-checklists/arrive2/info/#13) | Clearly describe the research question, research objectives and, where appropriate, specific hypotheses being tested. | Supplemental Material page 2-3 |
| Ethical statement | [#14](https://www.goodreports.org/reporting-checklists/arrive2/info/#14) | Provide the name of the ethical review committee or equivalent that has approved the use of animals in this study and any relevant licence or protocol numbers (if applicable). If ethical approval was not sought or granted, provide a justification. | Page 8 |
| Housing and husbandry | [#15](https://www.goodreports.org/reporting-checklists/arrive2/info/#15) | Provide details of housing and husbandry conditions, including any environmental enrichment. | No |
| Animal care and monitoring | [#16a](https://www.goodreports.org/reporting-checklists/arrive2/info/#16a) | Describe any interventions or steps taken in the experimental protocols to reduce pain, suffering, and distress. | No |
| Animal care and monitoring | [#16b](https://www.goodreports.org/reporting-checklists/arrive2/info/#16b) | Report any expected or unexpected adverse events. | No |
| Animal care and monitoring | [#16c](https://www.goodreports.org/reporting-checklists/arrive2/info/#16c) | Describe the humane endpoints established for the study, the signs that were monitored, and the frequency of monitoring. If the study did not set humane endpoints, state this. | Supplemental Material page 2-3 |
| Interpretation/scientific implications | [#17a](https://www.goodreports.org/reporting-checklists/arrive2/info/#17a) | Interpret the results, taking into account the study objectives and hypotheses, current theory, and other relevant studies in the literature. | Page 10-11 |
| Interpretation/scientific implications | [#17b](https://www.goodreports.org/reporting-checklists/arrive2/info/#17b) | Comment on the study limitations, including potential sources of bias, limitations of the animal model, and imprecision associated with the results. | Page 11 |
| Generalisability/translation | [#18](https://www.goodreports.org/reporting-checklists/arrive2/info/#18) | Comment on whether, and how, the findings of this study are likely to generalise to other species or experimental conditions, including any relevance to human biology (where appropriate). | Page 11, discussion |
| Protocol registration | [#19](https://www.goodreports.org/reporting-checklists/arrive2/info/#19) | Provide a statement indicating whether a protocol (including the research question, key design features, and analysis plan) was prepared before the study, and if and where this protocol was registered. | No |
| Data access | [#20](https://www.goodreports.org/reporting-checklists/arrive2/info/#20) | Provide a statement describing if and where study data are available. | Page 9 |
| Declaration of interests | [#21a](https://www.goodreports.org/reporting-checklists/arrive2/info/#21a) | Declare any potential conflicts of interest, including financial and nonfinancial. If none exist, this should be stated. | Page 24 |
| Declaration of interests | [#21b](https://www.goodreports.org/reporting-checklists/arrive2/info/#21b) | List all funding sources (including grant identifier) and the role of the funder(s) in the design, analysis, and reporting of the study. | Page 23 |

None The ARRIVE checklist is distributed under the terms of the Creative Commons Attribution License CC-BY. This checklist can be completed online using <https://www.goodreports.org/>, a tool made by the [EQUATOR Network](https://www.equator-network.org) in collaboration with [Penelope.ai](https://www.penelope.ai)
