# Supplementary material for: Combined Adaptive Immune Mechanisms Mediate Cardiac Injury After COVID-19 Vaccination
Source: Circulation. 2025 Oct 30;152(21):1485–500. doi: 10.1161/CIRCULATIONAHA.125.074644 (PMC12643570; doi:10.1161/CIRCULATIONAHA.125.074644)
Supplement: Supplementary file 2 [file cir-152-1485-s002.pdf]

Multiple immune mechanisms contribute to the development of post-Covid-19 vaccine myocarditis.
